# Association between Recent Falls and Changes in Outdoor Environments near Community-Dwelling Older Adults’ Homes over Time: Findings from the NHATS Study

**DOI:** 10.3390/ijerph16183230

**Published:** 2019-09-04

**Authors:** Sungmin Lee, Chanam Lee, Marcia G. Ory

**Affiliations:** 1 Department of Plant Science and Landscape Architecture, College of Agriculture, Health and Natural Resources, University of Connecticut, Storrs, CT 06268, USA; 2 Department of Landscape Architecture & Urban Planning, College of Architecture, Texas A&M University, College Station, TX 77843, USA; 3 Department of Environmental and Occupational Health, School of Public Health, Texas A&M Health Science Center, College Station, TX 77843, USA

**Keywords:** outdoor environmental characteristics, falls, elderly

## Abstract

Neighborhood environments have been increasingly associated with incidents of falling and the fear of falling. However, little is known about the causal impact of neighborhood environments on falling. This study identifies whether changes in outdoor environmental attributes over a one-year period are associated with the occurrence of recent falls among community-dwelling older adults aged 65 and older in the United States. Data were obtained from 4802 adults aged 65 years or older from the National Health and Aging Trends Study (NHATS). Logistic regression analyses were performed to identify neighborhood risk factors linked to the odds of experiencing recent falls at the one-year follow-up. Almost one in ten subjects (9.7% of 4802 subjects) who had not fallen before reported experiencing recent falls after one year. After adjusting for sociodemographic, health, and walking-related behavioral covariates, these subjects were more likely to reside in areas with higher environmental barriers on sidewalks/streets and uneven walking surfaces or broken steps, compared to non-fallers. Our findings suggest that safe and well-maintained outdoor environments may help prevent falls among community-dwelling older adults who engage in outdoor activities. Clinical and environmental interventions for promoting both safe walking and safe environments are warranted.

## 1. Introduction

Falling is especially prevalent among elderly individuals who engage in daily activities or walk around their neighborhoods [1]. Every year, one-fourth of people aged 65 years or older in the United States experience a fall, and one-fifth of falls cause serious injuries such as head trauma, broken bones, or hip fractures [2]. These fall-related injuries are substantial barriers to walking and healthy aging among older adults. Older adults who have had prior falls tend to experience restricted outdoor mobility, decreased physical activity, social withdrawal, and loss of confidence, prompting them to spend most of their time at home [3,4,5].

While there has long been an emphasis on multifaceted fall risks in terms of individual behavior and health [6], there is increasing interest in the relationship between the risk of falling and neighborhood environments. For example, the quality of the neighborhood, such as residential instability, threat of crime, social disorders, and physical hazards may affect not only the risk of falling but also the overall health outcomes throughout the lifespan [7,8,9,10]. In contrast, neighborhood social cohesion has been shown to be associated with decreased falls [11]. Moderate green space was also correlated to higher levels of physical activity and was found to be a protective factor against falls [12].

Neighborhood environments play a significant role in maintaining health and mobility among older adults given their increased physical and psychological vulnerability and mobility limitations as well as the changing use of space patterns that come with aging [13,14]. Decreases in physical and cognitive abilities also commonly result in older adults failing to cope with environmental obstructions (e.g., trash on the street), leading to injuries of varying severity [15]. Neighborhoods with low socioeconomic status and high physical disorders such as vacant buildings with many broken windows, crime, and generally poor maintenance conditions [16] may also be problematic and present fall risks for vulnerable older adults with visual or balance impairment. Extant research has also indicated that problems in walking surfaces such as uneven pavement and long crosswalks can directly influence the risk of falling [17].

Previous studies have shown that approximately half of the falls among community-dwelling older adults living in urban areas occur outdoors, often in the yard or on the street near their homes [18]. For example, various environmental conditions such as changes in ground level, uneven surfaces, and litter can increase the risk of falls in older adults. Recent studies have also provided supporting evidence that outdoor falls are heavily influenced by outdoor environmental risk factors [8,19]. In particular, Li et al. (2006) conducted a case-control study with a large sample and found that uneven and wet surfaces were associated with outdoor falls [18]. Later, Curl et al. (2016) developed an environmental audit checklist to assess the fall risk of outdoor environments and classified environmental causes of falls into changes in level, path condition and smoothness, path material, obstructions, road crossings, street lighting, and weather [20]. Qualitative studies have also shown that older individuals reported uneven walking surfaces, noise, inadequate maintenance, poor lighting, and unsafe traffic patterns as perceived risk factors [17,21].

While neighborhood environments have been increasingly recognized as important indicators of falling, limited longitudinal research has examined the causal relationship between changes in neighborhood environments and changes in falls over time. Falling is a maladaptive response when the demands of the environment exceed the individual’s functional ability [22]. In particular, falls occur when an individual faces unexpected environmental situations (icy or uneven surfaces, incomplete sidewalks) with a sudden loss of balance or gait change [23]. Because older adults are slower to respond to changes in their environment and can be influenced more by worsening environmental hazards compared to younger adults, it is important to understand how changes in built environments can lead to falls and fall-related injuries.

The objective of this 12-month prospective cohort study was to examine whether changes over time in outdoor environmental attributes were associated with a change from reporting no recent falls (at baseline) to reporting one or more recent falls (at follow-up) among older adults. We hypothesized that changes in outdoor environmental barriers near the home would be an important factor related to the increased odds of recent falls after controlling for variables such as sociodemographic characteristics, health, behavior conditions, and even home hazards. Specifically, we expected that those who encountered new environmental barriers or were consistently exposed to environmental barriers would face an increased likelihood of falling, compared to those who lived in places with fewer environmental barriers over time.

## 2. Methods

### 2.1. Study Design, Setting, and Sample

The data for the present study were taken from the National Health and Aging Trends Study (NHATS), which is an ongoing longitudinal study that surveys a nationally representative sample of 35.3 million Medicare beneficiaries aged 65 or older who reside in the United States [24]. The sample frame of NHATS relies on Medicare enrollment data and receives funding from the National Institute on Aging (NIA). Data were collected by trained personnel through in-person interviews and assessments. From 12,411 selected individuals, 8245 agreed to participate in the survey (weighted response rate = 71.6%) at baseline. At the 1-year follow-up, 6113 individuals who had responded in round one participated in the survey (weighted response rate = 84.9%) p [25]. Since our study sought to investigate the relationship between falling and outdoor environments near the home among community-dwelling residents, we excluded respondents who resided in nursing homes (*n* = 468) or similar care facilities (*n* = 412) and proxy respondents (*n* = 517), resulting in a sample of 6680 community-dwelling older adults at baseline. At the 1-year follow-up, 5659 of the 6680 respondents were interviewed again. We excluded those who had moved to non-community-dwelling housing (*n* = 332), resulting in 5327 participants. Finally, we excluded participants who reported never going outside (*n* = 36) at baseline because our study focus was on the risk of falling in outdoor environments, leaving 5291 participants for the final sample. Of the 5291 participants, 4802 (90.8%) reported no recent falls at baseline and so were included in the study analyses.

### 2.2. Dependent Variables: Occurrence of Recent Falls

Recent (within the previous month) fall incidents were self-reported by respondents in each survey period (baseline and follow-up). In each survey period, respondents were asked whether they had fallen in the previous month and whether they had fallen more than once (had one or more falls versus no falls). In this questionnaire, a “fall” referred to any slip or trip that caused the respondents to lose their balance and land on the floor, the ground, or another lower level. This definition is consistent with that of previous research from the Kellogg International Work Group (1987), which defined a fall as “an event which results in a person coming to rest inadvertently on the ground or other lower level” [26]. The occurrence of recent falls was operationalized in the longitudinal analysis as a change from reporting no recent falls at the time of the baseline interview to reporting one or more recent falls at the follow-up interview one year later. We used fall events during the previous one-month period as the outcome instead of yearly fall events because of better recall accuracy and consistency with the period of outdoor environmental measures. This approach potentially mitigated the temporal sequence of association.

### 2.3. Independent Variables: Outdoor Environmental Conditions

Independent variables for this study were the five outdoor environmental fall risk factors identified from previous literature [17,18,20]. The variables were classified into three domains: (a) environmental obstruction (litter or trash on sidewalks), (b) physical disorder (graffiti, vacant houses, or broken windows), and (c) problems related to walking surfaces (uneven walking surfaces or broken steps). These environmental conditions were derived from an environmental checklist completed by the interviewer before the in-person interview. The interviewer visually observed the outdoor environment around each participant’s home and marked the condition on the checklist. Each outdoor environmental barrier was specifically measured using a four-point scale (“none”, “a little”, “some”, and “a lot”) for three items: (1) the amount of litter, broken glass, or trash found on the sidewalks and streets; (2) the amount of graffiti on buildings and walls; and (3) the number of vacant/deserted houses or storefronts around the participant’s home. These variables were dichotomized for analysis (“none” versus “a little”, “some”, and “a lot”) because of non-normal distribution. In addition, two dichotomous responses (“no” versus “yes”) were included to measure perceived outdoor environmental conditions based on the following items: (4) whether there were many broken or boarded-up windows and (5) whether there were many uneven walking surfaces or broken steps around each participant’s home. Changes in these five items were then operationalized into three categories based on the changes in the follow-up measure compared to the baseline condition: (1) no environmental barriers at either observation or reduced barriers at follow-up; (2) increased barriers (no barriers at baseline but barriers at follow-up); and (3) barriers at both baseline and follow-up.

### 2.4. Covariate Measures

Several sociodemographic, health, and walking-related behavioral determinants of falling were identified based on previous studies, and these determinants served as covariates in this study [6,27]. For the sociodemographic dimension, we included age, sex, race/ethnicity (non-Hispanic White and others), marital status, and job status. The health dimension included obesity (normal: BMI < 25, overweight: 25 ≤ BMI < 30, and obese: BMI ≥ 30), self-reported health condition (excellent, very good, good, fair, and poor), fear of falling in the last month (yes or no), depression (“none” versus “several days of feeling depressed”, “more than half of the days of feeling depressed”, or “nearly every day of feeling depressed”), balance impairment in the last month (yes or no), and mobility limitations within a quarter-mile (yes or no). Vision impairment (yes or no) was also assessed based on three questions to determine whether participants (1) were legally blind; (2) had trouble reading newspaper print with glasses, contact lenses, or visual aids; and (3) were able to see a television across the room with the use of glasses or contact lenses. Three medical conditions (arthritis, stoke, and dementia), which are known to be highly associated with falls, were also included. For walking-related behaviors, we used two dichotomous questions (yes or no) about the use of a walking aid when traveling outside the home, walking to move to different outside locations in the last month, and the frequency of outside travel in the last month (0–4 days per week versus 5 or more days per week). Finally, we included home tripping hazards (e.g., broken lamps, flooring problems, or unclear pathways) to adjust for risk factors for indoor falls.

### 2.5. Statistical Analysis

The characteristics of the participants at baseline were reported using descriptive statistics. Differences in the distribution of sample characteristics between non-fallers and fallers were also investigated using the χ^2^ statistic. All reported responses were weighted by considering different probabilities of selection designed in NHATS. To understand the relationship between changes in outdoor environmental barriers and recent falls, we performed bivariate and sociodemographic-adjusted logistic regressions. Finally, we designed a fully-adjusted logistic regression model to investigate if outdoor environmental barriers remained significant in relation to the outcome variable (i.e., a change in fall incidents from baseline to follow-up) after controlling for individual sociodemographic, health, behavior, and home tripping hazards. A value of *p* < 0.05 was considered statistically significant for all analyses. We also used a change outcome (the occurrence of recent falls from baseline to follow-up) after adjusting for covariates in the longitudinal study to examine the prospective association between changes in the environmental perception and changes in fall occurrence. All statistical analyses were conducted using Stata IC 12.0 (Stata Corp, College Station, TX, USA).

## 3. Results

### 3.1. Participants’ Characteristics 

Table 1 shows the baseline characteristics of the participants. This study included only those who had no recent falls at baseline (*n* = 4802). On average, the participants were 76.5 years of age (SD = 7.3). Over half of the participants were female (56.8%) and non-Hispanic White (69.4%). Overall, 24.8% of the participants reported having a fear of falling. Although 53.6% reported having arthritis, 87.3% of the sample reported going outside five or more days a week. Among the sub-selected participants, 4337 participants (90.3%) had no recent falls a month prior to the follow-up interview, and 465 (9.7%) had recent falls at follow-up. Several significant differences were found between non-fallers and fallers between the baseline and follow-up interviews. Fallers were more likely to be in the oldest age group (over 85 years of age), have no job or retired, and have poor health, and have several health limitations (fear of falling, depression, balance impairment, mobility limitation, vision impairment, arthritis, and stroke).

### 3.2. Outdoor Environmental Factors Associated with Falls

Table 2 displays the likelihood of experiencing an increase in falls based on five outdoor environmental barriers for both the unadjusted analysis and partially adjusted analysis. Adjustments were made to the sociodemographic variables (age, sex, race, marital status, and job status). Two of the five environmental variables were significant in predicting the odds of experiencing falls at follow-up. Those who reported recent falls at follow-up were more likely than those who did not report recent falls at follow-up to be exposed to increased obstructions (litter/broken glass, or trash) on sidewalks or streets around their homes (OR = 1.81, 95% CI = 1.25–2.62) and to increased problems in walking surfaces (uneven walking surfaces or broken steps) over the two assessment periods (OR = 1.682, 95% CI = 1.25–2.27). In addition, those who were consistently exposed to litter or trash on the streets near their homes (OR = 1.88, 95% CI = 1.25–2.84) had an increased likelihood of reporting recent falls at follow-up.

### 3.3. Multivariate Analysis of Risk Factors for Falls 

Table 3 shows the odds ratios from the final multivariate model, which included the covariates and the two outdoor environmental factors that were significant (*p* < 0.05) in the previous and partially adjusted model. Several health factors were significantly associated with an increased risk of reporting recent falls at follow-up, including fair/poor health condition (OR = 1.52, 95% CI = 1.13–2.06), fear of falling (OR = 1.534, 95% CI = 1.19–1.97), depression (OR = 1.31, 95% CI = 1.04–1.66), balance impairment (OR = 1.32, 95% CI = 1.02–1.71), and mobility limitation (OR = 1.66, 95% CI = 1.23–2.24). Finally, two environmental variables continued to be significant in the full model. Compared to those who did not have an increase in fall incidents, those who reported recent falls during the follow-up assessment were more likely to live in areas with increased obstructions on the sidewalks or streets (OR = 1.57, 95% CI = 1.05–2.36) and with increased uneven walking surfaces or broken steps around their homes from baseline to follow-up (OR = 1.53, 95% CI = 1.11–2.14). In addition, environmental obstructions remained unchanged with some litter or trash on the street near their homes (OR = 1.68, 95% CI = 1.05–2.69). These obstructions were associated with increased odds of reporting recent falls at follow-up.

## 4. Discussion

We examined the associations between time-varying outdoor environmental measures and changes in the occurrence of recent falls. In this large cohort of older adults, the occurrence of recent falls was associated with changes in the outdoor environment near the subjects’ homes over time. In contrast to cross-sectional studies, our longitudinal analysis reveals the importance of changes in environmental conditions in relation to the occurrence of recent falls. After adjusting for individual sociodemographic, clinical, and indoor risk factors, this prospective study found that the prevalence of outdoor environmental barriers, such as obstructions on the street, were independently associated with a greater risk of falling among community-dwelling older adults. This finding is generally consistent with the findings of previous studies, showing that environmental obstructions on sidewalks and streets can cause imbalance and gait problems when an individual walks on the sidewalk [18,28]. It is also noteworthy that litter or trash on the street is suggestive of weak social control or lack of maintenance by residents, which, in turn, may cause older adults to feel unsafe walking outside [29]. Thus, our study suggests that safe and well-maintained outdoor environments may help prevent falls among those who frequently travel outside. A safe outdoor environment may encourage older adults to engage in health-promoting outdoor activities in the neighborhood.

Our study also found that participants who encountered new walking surface problems (uneven surfaces and/or broken steps) experienced an increased likelihood of reporting recent falls. This finding also aligns with previous studies, showing that unexpected changes in the walking level, such as uneven pavement or steps, influence the risk of falling [20]. We found that older adults who are consistently exposed to problems in outdoor walking surfaces at both baseline and follow-up did not change their risk of self-reported recent falls. This may be because older adults tend to adapt to their environment, enabling them to be cautious of consistent and predictable environmental risks, according to Lawton and Nahemow’s ecological theory of aging [30]. This association, however, warrants further investigation as the level of fall-related efficacy or environmental adaptation was not taken into account in this study. Instead, this finding suggests that environmental efforts to fix environmental barriers can help prevent falling.

The findings from this study, which identified significant links between outdoor environments and self-reported fall incidents, suggest the potential benefits of clinical, behavioral, and community-level intervention approaches. Because mobility options decrease with age, older adults tend to stay inside the home or engage in outdoor activities and walking near their home boundaries [31]. Thus, clinical screening and referrals to evidence-based multidimensional fall prevention programs are warranted for those who are at high risk of falling [32,33,34,35]. Physicians need to be aware of the role of the environment and take this factor into account when recommending increased exercise for their older patients [36]. Additionally, cautious monitoring of outdoor environments by both the older adults and their families could help prevent individual fall incidents. Finally, urban planning and city work departments can play an important role in promoting safe neighborhoods and maintaining the quality of sidewalks and street surfaces, thus reducing fall incidents and promoting health and quality of life among older adults.

Although the environmental assessments in this study were conducted in the areas immediately surrounding the participants’ dwellings without a specific definition or boundaries, future studies may adopt a life-space mobility approach, which is a pattern of areas defined by a distance extending from the location where a person sleeps to the rest of the dwelling. The area may include the garden, courtyard, or grounds surrounding the habitation; the block in which the dwelling is located; and the area across a traffic-bearing street [37]. To monitor where falls occur by location, a life-space model would help gerontologists and urban planners carefully predict the potential spots where fall incidents are most likely to occur. The evaluation could focus on mobility boundaries and mobility modes of older adults and their health conditions and then provide effective environmental modifications of the potential risky spaces. By adopting the life-space model, future studies could identify further environmental features that are associated with increased fall injuries based on individual mobility.

Future fall prevention research and intervention efforts should also consider modifiable environmental factors (improvement in street conditions and maintenance) together with other individually-oriented program interventions. These environmental interventions can help support the concept of “aging in place,” the ability to live in one’s own home or community as long as possible with a little attention to the physical environment [38]. Further, environmental improvements (e.g., fixing sidewalk surfaces) tend to last longer than many other program-based interventions [39]. In particular, they tend to be more effective and relevant to maintaining and protecting mobility and health of those who are currently active in their communities, and they can lead to lower healthcare costs and caregiving burden [40].

This study has both limitations and strengths in terms of the research design and data. First, the outdoor environmental variables were restricted to those collected in the NHATS survey, although we also used five outdoor environmental attributes captured from interviewer observations. Future studies may benefit from including additional outdoor environmental variables, including both perceived and objective measurements. We also used self-reported recent fall data for incidents that occurred within a month of the survey period to mitigate participant recall bias and to match the environmental variables measured at the time of the interview through interviewer observations. Such a temporal recall of recent fall incidents and changes in environmental attributes can weaken the causal inference. Further studies would benefit from using optimized methods to measure fall incidents and environmental changes more frequently and accurately, such as asking participants to keep fall diaries and environmental audit tools [41]. The use of singular questions to assess the controlled variables (e.g., depression or fear of falling) is a limitation as well.

Another limitation is that we were unable to include individual and neighborhood socioeconomic status in our analyses due to low response rates for the household income-related questionnaire and unavailability of geographic data for each participant’s dwelling. We were also unable to separate outdoor falls from general falls in our data. However, we added a covariate, indoor tripping hazards, to adjust for risk factors for indoor falls. To better understand the direct effects of the outdoor environment on fall incidents, researchers planning future studies and surveys are advised to include a separate questionnaire about the outdoor fall experience. Another limitation is the potential bias of evaluations made by different interviewers of the same outdoor environments. However, the items related to these environmental features (e.g., litter/trash on the streets or broken steps) were relatively straightforward.

Despite these limitations, our study has several important strengths. First, the study included a large sample, taken from a nationally representative sample of community-dwelling Medicare beneficiaries aged 65 years and older. Second, we accounted for a wide range of individual variables that explained increases in fall risk to better understand the association between changes in fall incidents and changes in outdoor environmental conditions over time. Third, to the best of our knowledge, this was the first study that investigated prospective evidence related to the effects of built environments on fall status. Thus, the current study makes a significant contribution to better understanding of the role of poor environmental conditions as fall risk factors. It also offers insights about relevant interventions that could reduce fall risk factors among community-dwelling older adults.

## 5. Conclusions

Walking is a popular form of meeting the guidelines for older adults’ recommended national physical activity to maintain healthy aging, and thus, walking should be encouraged by physicians [42,43]. However, given that walking is inherently related to fall incidents, it is important to support outdoor conditions immediately surrounding the home to enable older adults to walk around their neighborhoods without concern about falling. The findings from our study suggest that safe and well-maintained outdoor environments may help prevent falls among older adults who are currently active, independent of their underlying health conditions. Living conditions and the quality of the neighborhood environment have a strong impact on health. Thus, addressing the modifiable environmental risk factors of falling and maintaining safe outdoor environments near community-dwelling older adults may be important components of fall prevention efforts, in coordination with other clinical and behavioral risk factors.

## Figures and Tables

**Table 1:** Characteristics of study samples between non-fallers and fallers (*n* = 4802).

	Baseline	Follow-Up		*p*-Value
	N (%)	No Falls N (%)	Falls N (%)	
**Sociodemographic**				
Age				<0.001
65–74	2110 (43.9)	1930 (44.5)	180 (38.7)	
75–84	1951 (40.6)	1768 (40.8)	183 (39.3)	
85+	741 (15.4)	639 (14.7)	102 (21.9)	
Female	2729 (56.8)	2457 (56.7)	272 (58.5)	0.446
Non-Hispanic White	3335 (69.4)	3005 (69.3)	330 (71.0)	0.455
Never married, or divorced, widowed or separated	2331 (48.6)	2093 (48.3)	238 (51.2)	0.24
No job or retirement	4123 (86.8)	3708 (86.4)	415 (89.8)	0.041
**Health**				
Obesity				0.281
Normal (BMI < 25)	1587 (33.9)	1436 (34)	151 (33.3)	
Overweight (25 ≤ BMI < 30)	1762 (37.7)	1602 (37.9)	160 (35.2)	
Obese (BMI ≥ 30)	1329 (28.4)	1186 (28.1)	143 (31.5)	
Self-reported health condition				<0.001
Excellent-very good	2109 (43.9)	1964 (45.3)	145 (31.2)	
Good	1564 (32.6)	1419 (32.7)	145 (31.2)	
Fair-poor	1126 (23.5)	951 (21.9)	175 (37.6)	
Fear of falling in the previous month	1189 (24.8)	997 (23.0)	192 (41.4)	<0.001
Depression (Several days, more than half the days, nearly every day)	1238 (25.8)	1060 (24.5)	178 (38.3)	<0.001
Balance impairment in the previous month	1150 (23.9)	961 (22.2)	189 (40.6)	<0.001
Mobility limitation within a quarter mile in the previous month	1162 (24.3)	972 (22.5)	190 (41.3)	<0.001
Vision impairment	338 (7.0)	288 (6.6)	50 (10.7)	0.001
Arthritis	2568 (53.6)	2282 (52.7)	286 (61.6)	0.001
Stroke	449 (9.4)	377 (8.7)	72 (15.5)	<0.001
Dementia	92 (1.9)	78 (1.8)	14 (3.0)	0.07
Walking-related Behavior				
Use of walking aid to go outside in the previous month	1056 (22.0)	900 (20.8)	156 (33.6)	<0.001
Frequency of going outside in last month (5 or more days vs. 0–4 days)	4192 (87.3)	3808 (87.8)	384 (82.6)	0.001
**Indoor Environments**				
Tripping hazards (e.g., broken lamps, flooring problems, unclear pathways)	572 (12.7)	501 (12.3)	71 (16.1)	0.022
**Independent Variables (Time-Variant)**				
Litter/broken glass, or trash				0.001
No barriers at both periods or reduced barriers at follow-up	4252 (90.0)	3865 (90.6)	387 (84.9)	
Increased barriers at follow-up	258 (5.5)	220 (5.2)	38 (8.3)	
Barriers at both periods	214 (4.5)	183 (4.3)	31 (6.8)	
Graffiti on buildings and walls				0.114
No barriers at both periods or reduced barriers at follow-up	4566 (96.7)	4131 (96.8)	435 (95.2)	
Increased barriers at follow-up	119 (2.5)	104 (2.4)	15 (3.3)	
Barriers at both periods	39 (0.8)	32 (0.8)	7 (1.5)	
Vacant or deserted houses				0.748
No barriers at both periods or reduced barriers at follow-up	4139 (87.6)	3739 (87.6)	400 (87.7)	
Increased barriers at follow-up	213 (4.5)	190 (4.5)	23 (5.0)	
Barriers at both periods	372 (7.9)	339 (7.9)	33 (7.2)	
Broken window				0.114
No barriers at both periods or reduced barriers at follow-up	4303 (91.3)	3897 (91.5)	406 (89.0)	
Increased barriers at follow-up	125 (2.7)	107 (2.5)	18 (4.0)	
Barriers at both periods	283 (6.0)	251 (6.0)	32 (7.0)	
Uneven walking surfaces or broken steps				0.003
No barriers at both periods or reduced barriers at follow-up	4031 (85.6)	3665 (86.1)	366 (80.6)	
Increased barriers at follow-up	401 (8.5)	344 (8.1)	57 (12.6)	
Barriers at both periods	278 (5.9)	247 (5.8)	31 (6.8)	

Note: *p*-values based on Chi-squared test.

**Table 2:** Environmental predictors of recent falls at the 1-year follow-up in individuals without a history of falls: unadjusted analysis and partially adjusted analyses.

	Unadjusted Analysis			Adjusted Analysis ^a^		
	OR	(95% CI)	*p*-Value	OR	(95% CI)	*p*-Value
Litter/broken glass, or trash						
Increased barriers at follow-up	1.725 **	1.204–2.472	0.003	1.811 **	1.254–2.615	0.002
Barriers at both periods	1.692 *	1.140–2.511	0.009	1.883 **	1.247–2.844	0.003
Graffiti on buildings and walls						
Increased barriers at follow-up	1.370	0.790–2.375	0.262	1.384	0.793–2.417	0.253
Barriers at both periods	2.077 ^†^	0.912–4.734	0.082	2.304 ^†^	0.994–5.339	0.052
Vacant or deserted houses						
Increased barriers at follow-up	1.132	0.725–1.765	0.586	1.125	0.717–1.766	0.608
Barriers at both periods	0.910	0.627–1.320	0.619	0.937	0.638–1.374	0.737
Broken window						
Increased barriers at follow-up	1.615 ^†^	0.970–2.688	0.065	1.649 ^†^	0.988–2.751	0.056
Barriers at both periods	1.224	0.835–1.793	0.300	1.242	0.845–1.824	0.270
Uneven walking surfaces or broken steps						
Increased barriers at follow-up	1.659 **	1.229–2.240	0.001	1.682 **	1.245–2.274	0.001
Barriers at both periods	1.257	0.852–1.854	0.249	1.267	0.856–1.876	0.238

Note: ** *p* < 0.01, * 0.01 ≤ *p* < 0.05, ^†^ 0.05 ≤ *p* <0.1; ^a^: adjusted for sociodemographic variables (age, sex, race, marital status, and job status).

**Table 3:** Final multivariate model: falling risk factors.

	Total		
	OR	(95% CI)	*p*-Value
**Sociodemographic**			
Age (reference: 65–74)			
75–84	0.946	0.744–1.202	0.649
85+	1.326 ^†^	0.964–1.825	0.083
Female	0.963	0.763–1.215	0.749
Non-Hispanic White	1.281	0.997–1.644	0.052
Never married, or divorced, widowed or separated	0.866	0.688–1.09	0.219
No job or retirement	0.851	0.604–1.199	0.356
**Health**			
Obesity (Reference: Normal, BMI < 25)			
Overweight (25 ≤ BMI < 30)	1.076	0.834–1.388	0.575
Obese (BMI ≥ 30)	1.083	0.823–1.424	0.57
Self-reported health condition (Reference: Excellent-Good)			
Good	1.041	0.797–1.36	0.768
Fair-Poor	1.521 **	1.126–2.055	0.006
Fear of falling in the previous month	1.534 **	1.195–1.968	0.001
Depression	1.314 *	1.038–1.664	0.023
Balance impairment in the previous month	1.316 *	1.015–1.705	0.038
Mobility limitation within a quarter mile in the previous month	1.658 *	1.23–2.236	0.001
Vision impairment	1.143	0.801–1.633	0.461
Arthritis	1.024	0.814–1.288	0.841
Stroke	1.239	0.906–1.695	0.179
Dementia	1.385	0.752–2.553	0.296
Walking-related Behavior			
Use of walking aid to go outside in the previous month	0.894	0.662–1.207	0.464
Frequency of going outside in the previous month	1.215	0.889–1.661	0.222
**Indoor Environments**			
Tripping hazards (e.g., broken lamps, flooring problems, unclear pathways)	1.146	0.838–1.566	0.394
**Outdoor Environments**			
(reference: no barriers at both periods or reduced barriers at follow-up)			
Litter/broken glass, or trash			
Increased barriers at follow-up	1.574 *	1.052–2.356	0.027
Barriers at both periods	1.682 *	1.052–2.689	0.03
Uneven walking surfaces or broken steps			
Increased barriers at follow-up	1.538 *	1.109–2.135	0.01
Barriers at both periods	0.907	0.571–1.442	0.681

Note: ** *p* < 0.01, * 0.01 ≤ *p* < 0.05, ^†^ 0.05 ≤ *p* < 0.1; *n* = 4519; LR Chi2 = 151.74; *p*-value < 0.001.
